# Polycyclic Aromatic Hydrocarbons: Sources, Toxicity, and Remediation Approaches

**DOI:** 10.3389/fmicb.2020.562813

**Published:** 2020-11-05

**Authors:** Avani Bharatkumar Patel, Shabnam Shaikh, Kunal R. Jain, Chirayu Desai, Datta Madamwar

**Affiliations:** ^1^Post Graduate Department of Biosciences, UGC Centre of Advanced Study, Sardar Patel University, Anand, India; ^2^P. D. Patel Institute of Applied Sciences, Charotar University of Science and Technology, Anand, India

**Keywords:** toxicity, bioavailability, biostimulation, microbial remediation, integrated technologies

## Abstract

Polycyclic aromatic hydrocarbons (PAHs) are widespread across the globe mainly due to long-term anthropogenic sources of pollution. The inherent properties of PAHs such as heterocyclic aromatic ring structures, hydrophobicity, and thermostability have made them recalcitrant and highly persistent in the environment. PAH pollutants have been determined to be highly toxic, mutagenic, carcinogenic, teratogenic, and immunotoxicogenic to various life forms. Therefore, this review discusses the primary sources of PAH emissions, exposure routes, and toxic effects on humans, in particular. This review briefly summarizes the physical and chemical PAH remediation approaches such as membrane filtration, soil washing, adsorption, electrokinetic, thermal, oxidation, and photocatalytic treatments. This review provides a detailed systematic compilation of the eco-friendly biological treatment solutions for remediation of PAHs such as microbial remediation approaches using bacteria, archaea, fungi, algae, and co-cultures. *In situ* and *ex situ* biological treatments such as land farming, biostimulation, bioaugmentation, phytoremediation, bioreactor, and vermiremediation approaches are discussed in detail, and a summary of the factors affecting and limiting PAH bioremediation is also discussed. An overview of emerging technologies employing multi-process combinatorial treatment approaches is given, and newer concepts on generation of value-added by-products during PAH remediation are highlighted in this review.

## Introduction

Rapid industrialization and urbanization have resulted in numerous anthropogenic activities, which dump various pollutants in the environment, including polycyclic aromatic hydrocarbons (PAHs) (Mojiri et al., 2019). Due to their inherent properties, PAHs are persistent pollutants having a wide range of biological toxicity; remediation of PAHs from the environment has been a global concern. The PAH pollutants are ubiquitous, found equally in aquatic and terrestrial ecosystems as well as in the atmosphere (Adeniji et al., 2019). The rate of deposition of PAHs was found to accelerate in the soil/sediments due to their higher hydrophobicity and low aqueous solubility. They are strongly adsorbed onto soil particles, and therefore, the soil ecosystem becomes an ultimate sink for PAHs (Lu et al., 2011; Kuppusamy et al., 2017). Soil PAH pollution can be classified into three categories, i.e., unpolluted (∑PAH < 200 ng.g^–1^), weakly polluted (PAH 200–600 ng.g^–1^), and heavily polluted (PAH > 1,000 ng.g^–1^) (Wu et al., 2019).

The PAH pollution, either directly or indirectly, is strongly affecting the health and well-being of humans, along with other organisms across the planet (García-Sánchez et al., 2018). The choice of appropriate strategies for PAH remediation is always critical, as it is highly dependent on two major parameters: polluted matrix and environmental conditions (Kuppusamy et al., 2017). Different remediation methods involving physical, chemical, biological, and lately developed integrated approaches have been continuously applied at varying degree of success. Among the many remediation approaches, methods based on microorganisms for ecological restoration of PAH-polluted environments have been a well-evaluated approach (Kuppusamy et al., 2017; Malla et al., 2018; Mehetre et al., 2019). Recently, the integrated PAH remediation methods have also been reported for efficient mitigation of PAH pollutants. The aim of this review is to discuss current knowledge and recent developments in PAH remediation strategies, associated factors, and their effectiveness as well as limitations. This review also systematically outlines characteristics, sources, exposures, toxicity, and health effects of PAHs, as well as the significance of PAH remediation, insights of -omics approaches in PAH bioremediation, and constraints during PAH bioremediation.

## Polycyclic Aromatic Hydrocarbons: Physico-chemical Properties, Sources of Pollution, and Routes of Exposure

Polycyclic aromatic hydrocarbons are organic pollutants and composed of two or more fused aromatic rings of carbon and hydrogen atoms, which are primarily colorless, white, or pale yellow solid compounds (Abdel-Shafy and Mansour, 2016; Suman et al., 2016). The molecular arrangements of aromatic rings in space can be linear, angular, or in clusters (Abdel-Shafy and Mansour, 2016). With the number of rings present in the compounds, PAHs are classified into light-molecular weight PAHs (LMW PAHs; having two or three aromatic rings) and high-molecular weight PAHs (HMW PAHs; having four or more aromatic rings). Depending upon their molecular weight, they are emitted either as gaseous phase (LMW PAHs) or in the particulate form (HMW PAHs) (Lee and Vu, 2010). Further, based on the structure of rings, PAHs are also classified as: *alternant PAHs*, which contain only fusion of six carbon benzene rings, whereas the *non-alternant PAHs* like fluorene contain fusion of six carbon benzene rings along with an additional ring of less than six carbon (Gupte et al., 2016). The existence of dense π electrons on aromatic rings is responsible for the biochemical persistence of PAHs that make PAHs more resistant to nucleophilic attack (Haritash and Kaushik, 2009). The United States Environmental Protection Agency (USEPA) has declared 16 PAHs as priority pollutants in 1983 based on their existence of highest concentrations, greater exposure, recalcitrant nature, and toxicity (Zheng et al., 2018; Mojiri et al., 2019). PAHs are characterized through their low water solubility, low vapor pressure, and high melting and boiling points, depending on their structures (Lee and Vu, 2010). PAHs with increased molecular weight are tending to decrease water solubility and increase lipophilicity, making them more recalcitrant compounds (Okere and Semple, 2012). Table 1 shows the physicochemical properties of 16 PAHs.

**TABLE 1:** Physicochemical properties of 16 polycyclic aromatic hydrocarbons (Yan et al., 2004; Bojes and Pope, 2007; Ravindra et al., 2008; Lee and Vu, 2010; Ghosal et al., 2016).

Name	Formula	Structure	Molecular weight (g/mole)	Solubility in water (mg/L)	Phase distribution	Melting point (°C)	Boiling point (°C)	Vapor pressure (mmHg)	Log Kow	Log Koc	Toxicity as per IARC
Naphthalene	C_10_H_8_	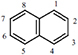	128.17	31	Gas	80.26	218	0.087	3.29	2.97	2B
Acenaphthene	C_12_H_10_	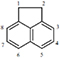	154.21	3.8	Gas	95	96	4.47 × 10^–3^	3.98	3.66	3
Acenaphthylene	C_12_H_8_	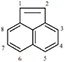	152.20	16.1	Gas	92–93	265–275	0.029	4.07	1.40	3
Anthracene	C_14_H_10_	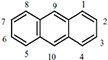	178.23	0.045	Particle gas	218	340–342	1.75 × 10^–6^	4.45	4.15	3
Phenanthrene	C_14_H_10_	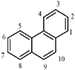	178.23	1.1	Particle gas	100	340	6.8 × 10^–4^	4.45	4.15	3
Fluorene	C_13_H_10_	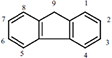	166.22	1.9	Gas	116–117	295	3.2 × 10^–4^	4.18	3.86	3
Fluoranthene	C_16_H_10_	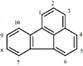	202.26	0.26	Particle gas	110.8	375	5.0 × 10^–6^	4.90	4.58	3
Benzo(a)anthracene	C_20_H_12_	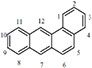	228.29	0.011	Particle	158	438	2.5 × 10^–6^	5.61	5.30	2B
Chrysene	C_18_H_12_	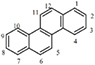	228.29	0.0015	Particle	254	448	6.4 × 10^–9^	5.9	No data	2B
Pyrene	C_16_H_10_	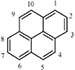	202.26	0.132	Particle gas	156	393–404	2.5 × 10^–6^	4.88	4.58	3
Benzo(a)pyrene	C_20_H_12_	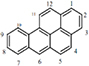	252.32	0.0038	Particle	179–179.3	495	5.6 × 10^–9^	6.06	6.74	1
Benzo(b)fluoranthene	C_20_H_12_	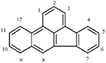	252.32	0.0015	Particle	168.3	No data	5.0 × 10^–7^	6.04	5.74	2B
Benzo(k)fluoranthene	C_20_H_12_	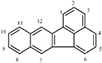	252.32	0.0008	Particle	215.7	480	9.59 × 10^–11^	6.06	5.74	2B
Dibenz(a,h)anthracene	C_22_H_14_	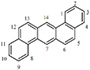	278.35	0.0005	Particle	262	No data	1 × 10^–10^	6.84	6.52	2A
Benzo(g,h,i)perylene	C_22_H_12_	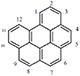	276.34	0.00026	Particle	273	550	1.03 × 10^–10^	6.50	6.20	3
Indeno[1,2,3-cd] pyrene	C_22_H_12_	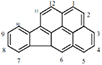	276.34	0.062	Particle	163.6	530	10^–10^–10^–16^	6.58	6.20	2B

*Kow is n-Octanol/Water Partition Coefficient. Log Kow is useful for predicting the distribution of compound in the environment, high Log Kow indicates low water affinity and high hydrophobicity. Compounds with Log Kow > 4.5 have higher bioaccumulation rates.*

*Koc is Soil Adsorption Coefficient. Log Koc is useful for predicting the mobility of compound in soil, i.e., distribution and exposure level of compound, high Log Koc indicates strong adsorption onto soil and organic matter. Compounds with Log Koc > 4.5 have potential adverse effects on terrestrial organisms.*

*Toxicity as per International Agency for Research on Cancer (IARC): group 1-human carcinogen, group 2A-probable human carcinogens, group 2B-possible human carcinogens, and group 3-not classifiable as human carcinogen.*

The sources of PAH pollution are categorized mainly into two, such as anthropogenic emission sources and natural emission sources (Mojiri et al., 2019). Natural emission sources such as volcanic eruptions, natural forest fire, and moorland fire caused by lightning flashes are negligible or less important (Srogi, 2007; Ravindra et al., 2008; Abdel-Shafy and Mansour, 2016). Anthropogenic emission sources are the main determinants of PAH pollution, which can be divided into four types, i.e., industrial, mobile, domestic, and agricultural emission sources (Ravindra et al., 2008). Incomplete combustion is the prime source of PAH emissions by various industrial activities such as waste incineration, iron and steel production, aluminum production, cement manufacturing, coal-tar pitch production, dye manufacturing, asphalt industries, rubber tire manufacturing, fungicide and insecticide production, exhaust from refineries, and power production (Srogi, 2007; Ravindra et al., 2008; Abdel-Shafy and Mansour, 2016; Gupte et al., 2016; Mojiri et al., 2019). Other industrial emission sources are coal gasification, electric arc furnace, oxygen furnace, diesel engine, and gasoline-powered engines of large machineries (Srogi, 2007; Ravindra et al., 2008). Mobile emission sources include exhaust from many vehicles like aircrafts, ships, trains, and off-road heavyweight and lightweight vehicles (Srogi, 2007; Ravindra et al., 2008).

**FIGURE 1:**
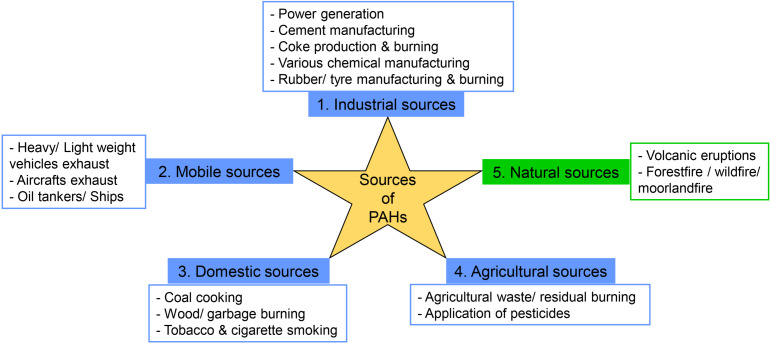
Different types of polycyclic aromatic hydrocarbon (PAH) emission sources: 1, 2, 3, and 4 belong to anthropogenic activities, and 5 belongs to natural activities.

Domestic emission sources involve household activities such as garbage burning, coal coking, wood burning, cooking on oil/gas burners and kerosene/wood stoves, and other residential heating (Johnsen and Karlson, 2007; Ravindra et al., 2008; Gupte et al., 2016). Agricultural emission sources are open biomass burning and agricultural waste burning when burning is performed under suboptimum combustion conditions (Ravindra et al., 2008). High PAH pollution in a rural area is mainly due to domestic and agricultural sources, whereas in an urban area due to industrial, mobile, and domestic sources. PAH concentration varies in all seasons; concentration is highest in winter followed by spring, autumn, and summer. The higher PAH level in winter and spring can be attributed to the high quantity of incomplete combustion of fossil fuel, elevated residential heating, lower photodegradation, and poor diffusion due to atmospheric conditions like calm winds and low temperature (Miura et al., 2019). Figure 1 shows different types of PAH emission sources.

The PAH sources are also categorized based on their origin of production into three types, i.e., pyrogenic, petrogenic, and biogenic (Mojiri et al., 2019). Pyrogenic PAHs are formed through unintentionally incomplete combustion of organic materials at very high temperatures (350–1,200°C) under no or low oxygen conditions (Abdel-Shafy and Mansour, 2016). Some intentional pyrolytic processes such as thermal breaking of petroleum complex compounds into lighter hydrocarbons and distillation of coal into coal tar and coke also produce pyrogenic PAHs. The concentrations of pyrogenic PAHs are generally higher in urban areas (Abdel-Shafy and Mansour, 2016; Mojiri et al., 2019). Petrogenic PAHs are present in petroleum and its by-products that are widespread due to storage, transport, use, and leakage of crude oil and its products (Abdel-Shafy and Mansour, 2016). Pyrogenic sources are predominated by HMW PAHs, and petrogenic sources consist majorly of LMW PAHs (Marris et al., 2020). Biogenic PAHs are synthesized by biological entities like microorganisms, phytoplankton, algae, and plants and during slow biological conversion of organic materials (Mojiri et al., 2019).

Atmospheric PAHs (gaseous phase as aerosols) are deposited in water, soil, and plants in the particulate phase through dry/wet deposition processes (Abdel-Shafy and Mansour, 2016). PAHs with three or more aromatic rings are very strong adsorbent to the soil particles due to low vapor pressure and high hydrophobicity (Abdel-Shafy and Mansour, 2016). Accumulation of PAHs in soil/sediment is responsible for further transport of pollution to the groundwater, plants, and food, e.g., plant roots absorb PAHs from polluted soil and translocate to the farther plant parts. Exposure to PAHs is unavoidable in the current situation. PAH exposure occurs mainly *via* three routes, i.e., inhalation, ingestion, and dermal contact (Burchiel and Luster, 2001). It is also possible that exposure can occur *via* more than one route, simultaneously, e.g., dermal and inhalation exposures from contaminated soil (Rengarajan et al., 2015; Abdel-Shafy and Mansour, 2016). For many people, the primary exposure occurs at the workplace, e.g., workers in coke manufacturing factories and food processing industries, traffic police through inhalation of vehicle exhaust and road dust containing PAHs (Lee and Vu, 2010). All are not workplace-based exposure, such as consuming polluted water, grilled and smoked food items, smoking, etc. (Lee and Vu, 2010; Suman et al., 2016). For smoking people, one cigarette causes an intake of 20–40 ng of benzo(a)pyrene (Skupinska et al., 2004). Up to 70% of PAH exposure can be related to diet for non-smoking persons (Skupinska et al., 2004).

In drinking water, PAH concentration varies between 1 ng/L and 11 μg/L, and the highest acceptance by WHO for benzo(a)pyrene is 0.7 μg/L (Skupinska et al., 2004). PAHs are formed during domestic and industrial food processing like roasting, toasting, drying, grilling, frying, baking, and barbecuing (Rose et al., 2015). Vegetables and fruits may be contaminated by their growth in PAH-polluted soil and atmospheric deposition (Zelinkova and Wenzl, 2015). The world’s two most popular beverages, tea and coffee, are also contaminated with PAHs through atmospheric deposition on raw plants, industrial drying/roasting processes, and heating steps in preparation (Duedahl-Olesen et al., 2015). Duedahl-Olesen et al. (2015) investigated the presence of PAH4 [chrysene, benzo(a)pyrene, benzo(a)anthracene, benzo(b)fluoranthene] in 18 brands of tea and 13 brands of coffee. The highest PAH4 was detected in black tea (25–115 μg/kg) and in instant coffee (2.2–5.1 μg/kg).

As per report of the German Environment Agency, PAHs are found in using products, e.g., toys, bathing shoes, mouse pads, bicycle handles, many sports items, etc., daily (Brandt and Einhenkel-Arle, 2016). The desired elasticity of rubber-made products and softness and flexibility in polyvinyl chloride (PVC)-made products are achieved using tar oils, extender oils, and industrial soots, which have extended PAHs. The PVC bathing shoes were found to have 546 mg/kg of 16 PAHs, among all, fluorene and phenanthrene levels were highest, 170 and 120 mg/kg, respectively. Paschke et al. (2015) detected benzo(a)pyrene, pyrene, phenanthrene, and naphthalene in newspaper’s ink at levels up to 52, 553, 778, and 283 μg/kg, respectively. PAHs are inevitable in life even though they have toxic effects because of their presence in daily usable products including foodstuffs.

## Biotoxicity of Noxious Polycyclic Aromatic Hydrocarbon Pollutants

Individual PAH compounds do not have the exact same health effects (Rengarajan et al., 2015; Abdel-Shafy and Mansour, 2016). Many PAHs are mutagenic, carcinogenic, teratogenic, and immunotoxic to living organisms, including microorganisms, animals, and humans (Burchiel and Gao, 2014; Rengarajan et al., 2015; Bolden et al., 2017). PAHs have ecotoxic effects on aquatic life and birds (Abdel-Shafy and Mansour, 2016). The mode of exposure, exposure duration, and exposure dose are important parameters for the severity of PAHs’ toxic effects (Rajpara et al., 2017; Tong et al., 2018). Zheng et al. (2018) found that PAHs from different sources showed different risk levels, and they calculated incremental lifetime cancer risk (ILCR) in humans for soil-bound PAHs upon the three different exposure routes; the highest cancer risk was found for ingestion, i.e., 98.1–99.3%, followed by dermal contact, i.e., 0.66–1.83%, and inhalation, i.e., 0.03–0.04%. Toxic effects of PAHs may vary according to factors such as pre health status and age. Acute health effects include eye irritation, vomiting, diarrhea, confusion, skin irritation, and inflammation (Abdel-Shafy and Mansour, 2016). Naphthalene, anthracene, and benzo(a)pyrene are direct skin irritants and skin sensitizers for animals and humans (Rengarajan et al., 2015). Chronic health effects include eye cataracts, kidney and liver damages, breathing problems, decreased immune function, lung malfunctions, and asthma-like symptoms (Abdel-Shafy and Mansour, 2016). Naphthalene can cause the breakdown of red blood cells if inhaled or ingested in high amounts (Rengarajan et al., 2015).

### Phototoxicity of Polycyclic Aromatic Hydrocarbons

Sunlight has three components, i.e., 91.0% visible light (400–700 nm), 8.7% UVA light (320–400 nm), and 0.3% UVB light (280–320 nm) (Yan et al., 2004). PAHs can absorb UVA and visible light. Due to the absorption of UVA light, formation of reactive species upon electron or energy transfer from the excited PAHs and formation of reactive intermediates upon the reaction of excited PAHs with oxygen or other molecules occur in the cells. These reactive species or intermediates are responsible for damages to cellular components such as cell membrane, nucleic acid, or proteins. PAH-contaminated human skin exposed to sunlight irradiation can cause DNA single-strand cleavage, oxidation of DNA bases, and formation of DNA covalent adducts, indicating PAH toxicity can exceed over 100 times in the presence of light as compared to dark (Yu, 2002; Yan et al., 2004).

### Genotoxicity and Carcinogenicity

Polycyclic aromatic hydrocarbon detoxification in mammalian systems occurs mainly in the liver *via* catalytic reactions of cytochrome P450 and many oxidase enzymes by generating water-soluble epoxide glutathione conjugates (Yu, 2002; Abdel-Shafy and Mansour, 2016). However, metabolism of some PAHs also generates reactive intermediates (e.g., diolepoxides, quinones, hydroxyalkyl derivatives), which are not sufficiently polar to be excreted, they form covalent adducts with nucleic acid and lead to genotoxic effects (Abdel-Shafy and Mansour, 2016). The International Agency for Research on Cancer (IARC) has categorized PAHs in four groups, i.e., group 1 as carcinogenic to humans, group 2A as probably carcinogenic to humans, group 2B as possibly carcinogenic to humans, and group 3 as not classifiable as carcinogenic to humans (Ghosal et al., 2016; Table 1). Benzo(a)pyrene is considered one of the most carcinogenic PAHs and generally used as an exposure marker for risk assessments (Lee and Vu, 2010). Tong et al. (2018) suggested that there is a 45% possibility of carcinogenic risk if PAH exposure exceeds the acceptable threshold (10^–6^). Significant accumulation and bioavailability of PAHs in the internal organs, which are rich in adipose tissue, after exposure are due to the high lipophilicity (Lee and Vu, 2010; Abdel-Shafy and Mansour, 2016). Several organs susceptible to tumor formation due to long-term exposure to PAHs include lung, skin, esophagus, colon, pancreas, bladder, and women’s breast (Yu, 2002; Rajpara et al., 2017).

### Teratogenicity and Human Reproductive System Abnormalities

Embryo toxicity has been reported in experimental animals due to exposure to naphthalene, benzo(a)anthracene, and benzo(a)pyrene (Rengarajan et al., 2015; Abdel-Shafy and Mansour, 2016). As per the scoping review by Bolden et al. (2017), these compounds may act as antiestrogens and/or antiandrogens by directly binding with estrogen and androgen receptors. Bolden et al. (2017) noted many non-cancer reproductive system-related health effects in both males and females due to PAH exposure, such as changes in sperm quality, testicular function, and egg viability, as well as DNA damage in oocytes, ovarian damages, and other reproductive diseases. The human studies evaluated affected health outcomes like polycystic ovary syndrome, fertility, spontaneous abortion, and premature birth.

The important hormonal regulators of reproduction such as luteinizing hormone, follicle-stimulating hormone, gonadotrophin-releasing hormone, and aromatase enzyme may be impacted by PAHs (Bolden et al., 2017). Smith et al. (2007) explained that benzo(a)pyrene induced infertility in the male reproductive system. Children do not require higher exposure for the same adverse health effects as adults. Children are prone to behaviors for increased PAH exposure, e.g., eating polluted soil, crawling on bare dirt surfaces, and many more hand–mouth activities. The Center for Children’s Environmental Health reports demonstrate that exposure of PAHs during pregnancy is responsible for adverse birth outcomes like low weight, premature delivery, and heart malformations (Rengarajan et al., 2015). High prenatal PAH exposure is connected to a low IQ and increased behavioral problems in the early-age child and childhood asthma (Perera et al., 2014; Rengarajan et al., 2015).

### Immunotoxicity of Polycyclic Aromatic Hydrocarbons

Polycyclic aromatic hydrocarbons have some immune system-related adverse effects like inhibition of pre B, pre T, and myeloid cell development, B and T cell suppression, apoptosis of lymphoid tissues, disruption of myelopoiesis, and altered cytokine production by macrophages and monocytes (Burchiel and Luster, 2001; Burchiel and Gao, 2014; Rengarajan et al., 2015). Under specific circumstances, tumor development, hypersensitivity (allergy), and autoimmunity may develop (Abdel-Shafy and Mansour, 2016). PAHs bind with specific aryl hydrocarbon receptors (AhRs) in lymphocytes and accessory cells of the immune system, upregulate AhR-controlled metabolic enzymes cytochrome P450, and produce immune toxic oxidative and electrophilic metabolites (Burchiel and Gao, 2014; Marris et al., 2020). The required PAH concentration to suppress humoral and cell-mediated immunity in mice is extremely high, 10–50 mg/kg of benzo(a)pyrene (Burchiel and Gao, 2014). Exposure to PAHs alters the structural and functional changes in bone marrow, which cause crucial health consequences, as bone marrow is the central organ of hematopoiesis and vital site for the production of immune system cells (Hrudkova et al., 2004). The required PAH concentration is higher to produce immunotoxicity than to produce cancer (Burchiel and Gao, 2014).

## Analysis of Polycyclic Aromatic Hydrocarbon Pollutants in Environmental Samples

Analytical techniques for PAH characterization in environmental samples involve extraction and detection techniques. Drawbacks of conventional solid-phase extraction and liquid–liquid extraction methods are overcome by advanced extraction methods, mainly classified as microextraction methods and miniaturized extraction methods (Manousi and Zachariadis, 2020). Microextraction methods include various solid-phase microextraction (SPME) (e.g., flow injection and syringe SPME) and various liquid-phase microextraction (LPME) (e.g., single-drop and hollow-fiber LPME, dispersive liquid–liquid microextraction, ultrasound/vortex-assisted LPME). Miniaturized extraction methods include dispersive solid-phase extraction, stir bar/rod/plate sorptive extraction, magnetic solid-phase extraction, fabric-phase sorptive extraction, and pipette tip solid-phase extraction. PAH extraction is improved by implementation of novel sorbents in advanced extraction methods like metal-organic frameworks, zeolitic imidazole frameworks, graphene, carbon nanotubes, graphene oxide, and molecularly imprinted polymers. Hazardous organic solvents are replaced with ionic liquids in LPME. Benefits of advanced extraction methods are simplicity, less time-consuming steps, less solvent requirements, lower volume samples, and easy handling (Manousi and Zachariadis, 2020). The most applicable detection methods are high-performance liquid chromatography (HPLC), gas chromatography (GC), gas chromatography-mass spectrometry (GC-MS), and ultra-high-pressure LC along with UV detector, diode-array detector, tandem-mass detector, flame ionization detector, and fluorescence detector (Manousi and Zachariadis, 2020).

## Physical and Chemical Methods for Polycyclic Aromatic Hydrocarbon Remediation

Remediation strategies implicate the reduction of pollutants in the environment until safe levels are attained by degradation or transformation processes in air, water, and soil. Pollution caused by PAHs is an onerous global concern due to their adverse effects. To restore the environment from PAH pollution, various remediation strategies have been employed including physical, chemical, and biological methods.

Due to the hydrophobic property of PAHs, they are soluble in organic solvents like acetone, alcohol, hexane, dichloromethane, methyl ethyl ketone, toluene, etc. Therefore, the use of suitable individual solvents or a mixture of solvents can be used to remove PAHs from water, sludge, and soil (Peng X. et al., 2018). *Soil washing with solvent* is a suitable approach for the removal of HMW PAHs, which are difficult to remove from the soil due to their low bioavailability and strong affinity to soil (Kuppusamy et al., 2017). Non-toxic and biodegradable extraction agents, e.g., cyclodextrins, vegetable oil, humic acid, supercritical and subcritical fluids, can also be used in the soil washing method (Gan et al., 2009). Solvent regeneration can be possible by distillation with the approximate loss of 10% solvent. Soil washing is recognized as a highly efficient method, which can be integrated along with other methods to achieve maximum degradation (Gitipour et al., 2018). Use of surfactant may enhance the efficiency of soil washing by changing the solubility of PAHs. *Surfactant-aided soil washing* was strongly dependent on PAH properties, surfactant structure, and soil composition (Gan et al., 2009; Gitipour et al., 2018).

Various *membrane-based filtration* methods such as ultrafiltration, micro/nano-filtration, and reverse osmosis can be applied for PAH removal from water (Smol and Włodarczyk-Makuła, 2012; Smol et al., 2016; Li et al., 2019). Gong et al. (2017) investigated electrocoagulation integrated membrane filtration approach for PAH removal from paper making wastewater (PMWW) to achieve water quality that is reusable, and they reported 94% of PAH removal from electrocoagulated PMWW using low-pressure reverse osmosis membranes at 25°C. Several *adsorbents* like activated carbon, charcoal, biochar, modified clay, magnetic nanomaterials, graphene oxide, nano-sulfonated graphene (SGE), and electro spun nanofibers have been used to remove PAHs from water and soil (Lamichhane et al., 2016; Xinhong et al., 2017).

Xinhong et al. (2017) selected nano-SGE over Tween 80 and methyl-β-cyclodextrin as *in situ* soil washing agent for PAH removal from coking plant soil, and they reported 80% of PAH removal under optimum washing conditions, which were 2,000 mg.L^–1^ concentration of SGE, 10:1 ratio of liquid/soil, and four cycles of successive washing. SGE properties such as strong adsorption capabilities for pollutants, high surface area for adsorption, high dispersibility, and separate deposition during centrifugation with soil make an ideal soil washing agent (Xinhong et al., 2017). Biochar has better adsorption for HMW PAHs than LMW PAHs because biochar needs a very high temperature for activation, at which LMW PAHs may be converted into gaseous phase (Peng X. et al., 2018). The PAH removal rate is highly affected by factors like reaction temperature, pH, humidity, concentration of adsorbent, etc. (Peng X. et al., 2018).

*In situ electrokinetic remediation* can be applied to PAH-polluted soils having less hydraulic conductivity by applying direct low-intensity electric current through electrodes (Kuppusamy et al., 2017). This method is more efficient with solubilizing agents of PAHs such as solvents, surfactants, vegetable oil, and cyclodextrins and more advantageous when it is applied with other *in situ* biodegradation methods (Kuppusamy et al., 2017). Li et al. (2020) assessed electro-bioremediation approach for pilot-scale PAH removal from polluted soil of an abandoned coking plant site. Degradation of total PAHs was achieved – 69% by electro-bioremediation approach as compared to 29% by only bioremediation after 182 days of incubation (Li et al., 2020). *Thermal technologies* include incineration of PAHs from polluted soil and industrial wastes at high temperatures (900–1,200°C) that destroy or volatilize the PAHs (Gan et al., 2009; Kuppusamy et al., 2017). *In situ* incineration is quite safe due to slight or no PAH emission into the atmosphere during heating because it uses a vacuum system or carrier gas for sweeping the volatile PAHs in the attached gas treatment arrangement for secondary removal of PAHs (Kuppusamy et al., 2017). The major disadvantage of incineration technology is a high energy requirement that makes it costly.

Most physical methods simply transfer PAHs from water and soil, but there are no structural changes in PAHs; therefore, physical methods cannot completely remove PAHs from the environment. Due to time-consuming and inefficient role of physical methods for PAH removal, chemical methods have been gaining attention (Peng X. et al., 2018). The most common approach for PAH removal by chemical methods is *oxidation processes* using common oxidants like ozone (O_3_) and Fenton reagent (Fe^2+^ + H_2_O_2_) as well as other oxidants like potassium permanganate (KMnO_4_), peroxy-acid (R-COOH), hydrogen peroxide (H_2_O_2_), and activated sodium persulfate (Na_2_S_2_O_8_) (Ukiwe et al., 2013; Das and Das, 2015; Peng X. et al., 2018). During oxidation through the Fenton reagent, unstable hydroxyl radicals (•OH) are generated in the presence of ferrous iron (Fe^2+^), which are utilized to degrade PAHs either by hydrogen abstraction or by hydroxyl addition (equations 1–3) (Gong et al., 2017). The requirements of acidic pH (2.8–3.0) during the oxidation process and ferric iron (Fe^3+^) removal after oxidation are the disadvantages, which make Fenton oxidation impractical and expensive as well as disturbing soil quality and soil microbes (Mojiri et al., 2019). These can be overcome by applying modified Fenton oxidation at neutral pH using chelating agents (capable of maintaining iron in dissolved form) along with Fenton reagents such as cyclodextrins, EDTA, citric acid, humic acid, oxalic acid, catechol, etc. (Usman et al., 2016).

(1) $${\text{H}}_{2}{\text{O}}_{2}+{\text{Fe}}^{2+}\to •\mathrm{OH}+{\mathrm{OH}}^{-}+{\text{Fe}}^{3+}$$

(2) $$\text{RH}+•\text{OH}\to •R+H_{2}\text{O}$$

(3) R+•OH→•ROH

The efficiency of ozone oxidation is strongly dependent on the water content of PAH-polluted soil; increased water content has an adverse effect since PAHs become less accessible to ozone due to occupied pore spaces by water (Gan et al., 2009). HMW PAHs are less susceptible to ozone and Fenton reagent oxidation due to their stronger affinities with humic acid of soil (Gan et al., 2009). The use of ultraviolet radiation or addition of other oxidants along with ozone can improve the oxidation rate (Ukiwe et al., 2013). Limitation of low aqueous solubility and low vapor pressure of PAHs during chemical degradation can be overcome by adding surfactants (Ukiwe et al., 2013; Gupte et al., 2016). The activation (heat, alkaline, and iron) aids to achieve better results than original oxidation treatments (Peluffo et al., 2018). Song et al. (2019) investigated PAH removal at *in situ* pilot-scale by different Zero-valent iron (ZVI)-activated persulfate oxidations and found PAH removal efficiencies 82, 63, and 69% by nanostructured ZVI, stearic-coated nanostructured ZVI, and micron-sized ZVI-activated persulfate oxidations, respectively, within 104 days of treatments.

Zhao et al. (2013) showed that thermal activation at 60°C and use of citrate chelated ferrous iron (Fe^2+^) along with persulfate oxidation enhanced HMW and total PAH removal (99 and 83%) as compared to control (persulfate oxidation without activation and addition) in the polluted soil of the coking plant due to generation of more powerful and stable persulfate (•SO_4_^1–^) and hydroxyl radicals (•OH). The oxidation process is affected by various factors like temperature, pH, divalent iron ion concentration, hydrogen peroxide concentration, oxidant selection, dose of oxidants, age of polluted soil, soil type, etc. (Lemaire et al., 2013). Addition of insufficient chemical oxidants results in dead-end products that are resistant to further degradation. The dead-end products become the source of further pollution if biological remediation for them is not available (Ukiwe et al., 2013).

*Photocatalysis* is an important approach to remove PAHs from the environment. Photocatalytic materials such as titanium dioxide (TiO_2_), zinc oxide (ZnO), silicon dioxide (SiO_2_), etc., can be used with ultraviolet light (Jiang et al., 2018). Titanium dioxide is the most effective photocatalytic material for removal of PAHs because of its higher oxidability and stability (Wen et al., 2003). Photochemical PAH degradation involves some oxidative species, which are produced during chemical oxidation of PAHs (Ukiwe et al., 2013).

## Bioremediation of Polycyclic Aromatic Hydrocarbon Pollutants

Biological methods are environment-friendly and have gained a lot of attention for PAH remediation recently due to several drawbacks of physical and chemical methods including cost, procedural complexity, regulatory burden, and lack of complete degradation (Agnello et al., 2016; Ghosal et al., 2016). Bioremediation techniques are broadly classified as *ex situ* or *in situ*. *Ex situ* techniques involve the physical removal of the contaminated materials (soil: excavation and water: pumping) to another area possibly within the site for treatment. *In situ* techniques involve treatment of the contaminated material in place, which have great public consent due to less expense, minimal site interruption, and high possibility of permanent waste elimination (Shah, 2014).

### Microbial Remediation of Polycyclic Aromatic Hydrocarbon Pollution

As per Kuppusamy et al. (2017), biological methods have gained wide attentions for PAH remediation, followed by integrated methods, chemical oxidation, and physical methods. Among all biological methods for PAH remediation, usage frequency of natural attenuation, bioaugmentation, and biostimulation is highest (∼33%), followed by bioreactors (22%), phytoremediation/rhizoremediation (22%), composting (13%), biopiles (4%), enzyme-mediated bioremediation (2%), vermiremediation (2%), and others (2%) (Kuppusamy et al., 2017). Moreover, the second most attractive integrated methods are applied in the subsequent order, i.e., biological–biological (42%), chemical–biological (27%), physical–chemical (21%), physical–chemical–biological (5%), and thermal–chemical methods (5%) (Kuppusamy et al., 2017). Microbial PAH remediation (bioaugmentation and biostimulation) deals with separate or combined application of specific microbes such as bacteria, archaea, fungi, and algae. However, bacteria- and fungi-assisted degradation has been widely studied.

#### Bacteria

Bacteria have unique metabolic versatility for degradation of PAH pollutants (Ma and Zhai, 2012). During bacterial aerobic PAH degradation, the oxygen works as the final electron acceptor and also as a co-substrate for the hydroxylation and oxygen-mediated cleavage of the aromatic ring (Chen et al., 2016), whereas bacterial anaerobic PAH degradation utilizes an entirely diverse approach to break and open the aromatic ring depending on the reductive reaction type and alternative final electron acceptors (Ghosal et al., 2016; Dhar et al., 2020). Chiefly, the bacteria perform aerobic PAH degradation using oxygenase-facilitated metabolism (comprising monooxygenase and dioxygenase enzymes). The first step in the aerobic PAH degradation is the hydroxylation of the aromatic ring through dioxygenase enzymes and formation of the cis-dihydrodiol, which ultimately oxidized to diol intermediates with the help of dehydrogenase enzymes.

These diol intermediates finally break open through the action of intra diol or extra diol ring-breaking dioxygenases *via* either ortho-cleavage or meta-cleavage pathway, able to form intermediates such as catechol, gentisic acids, and protocatechuic acid, which finally transform to tricarboxylic acid (TCA) cycle intermediates (Mallick et al., 2011). Dioxygenase is the multi-enzyme complex usually comprising of reductase, ferredoxin, and terminal oxygenase subunits (Mallick et al., 2011; Ghosal et al., 2016; Wu et al., 2020). Bacteria also strategize PAH degradation by the cytochrome P450-assisted pathway with the formation of trans-dihydrodiols or anaerobically under nitrate- and sulfate-reducing conditions (Lu et al., 2011; Mallick et al., 2011; Yang et al., 2020). Although aerobic PAH degradation is conventional and preferable, anaerobic PAH degradation is gaining more attentions nowadays due to the presence of anoxic conditions in diverse environmental niches such as phreatic zone, deep aquatic sediment, and water-flooded soil (Ghosal et al., 2016; Dhar et al., 2020).

The aerobic and anaerobic bacterial species have been reported extensively in the literature for the degradation of LMW and HWM PAHs through pure cultures, consortia, and mixed bacterial culture approaches (Table 2). The enhanced or complete PAH degradation can be achieved by mixed bacterial cultures and bacterial consortia as results of collaborative catabolic activities of participants and possibly presence of diverse degradation pathways. Therefore, most of the recent studies emphasized on mixed bacterial culture- and consortia-assisted PAH degradation (Vaidya et al., 2018; Haleyur et al., 2019; Patel et al., 2019). Degradation by immobilized bacteria and genetically modified bacteria is also a considerable approach (Peng X. et al., 2018). One of the major difficulties for degradation in soil/sediment is a dispersion of inoculum; it is easy for surface soil, however, challenging for subsurface soil due to limited microbial transport as cells adhere strongly to soil organic matter.

**TABLE 2:** Polycyclic aromatic hydrocarbon biodegradation under aerobic and anaerobic conditions using bacteria, archaea, fungi, algae, and co-cultures.

Inoculum	PAHs used in the study	Degradation condition	Degradation (%)	References
**Bacteria**				
Mixed bacterial cultures DAK11: *Pseudomonas aeruginosa* DAK11.1, *Pseudomonas stutzeri* DAK11.2, *Achromobacter* sp. DAK11.3, and *Chelatococcus* sp. DAK11.4	Naphthalene, Phenanthrene, Fluoranthene, and Pyrene	Liquid medium Aerobic	75, 86, 76, and 76	Patel et al., 2018
Immobilized *Pseudomonas taiwanensis* PYR1 and *Acinetobacter baumannii* INP1 on cinder beads	Pyrene and Indeno[1,2,3-cd]pyrene	Petroleum-contaminated soil Aerobic	71 and 81%	Huang et al., 2016
Bacterial community	16 Priority PAHs	*In situ* Windrows of 5,000 tons polluted soil Aerobic	85% total PAHs	Lors et al., 2012
Microbial community associated with anaerobic sediment	16 Priority PAHs (with nitrate and sulfate)	Sediment Anaerobic	37, 21, and 28%	Yang et al., 2020
**Extremophiles**				
Halophilic consortia Qphe-SubIV *Halomonas* strain and unculturable strain belonging to the genus *Marinobacter*	Phenanthrene	Liquid medium (5% NaCl) Aerobic	>90%	Dastgheib et al., 2012
Acidophilic *Stenotrophomonas maltophilia* AJH1	Anthracene, Phenanthrene, Naphthalene, Fluorene, Pyrene, Benzo(e)pyrene, and Benzo(k)fluoranthene	Liquid medium (pH 2) Aerobic	91, 90, 96, 95, 86, 82, and 79%	Arulazhagan et al., 2017
Thermophilic Mix culture: *Aeribacillus pallidus* U2, *Bacillus axarquiensis* UCPD1, *Bacillus siamensis* GHP76, and *Bacillus subtilis* subsp. *inaquosorum* U277	Anthracene, Fluorene, Phenanthrene, and Pyrene	Liquid medium At 50°C Aerobic	96, 86, 54, and 71%	Mehetre et al., 2019
**Archaea**				
Indigenous halophilic archaean *Haloferax elongans*, *Halobacterium noricense*, *Haloferax larsenii*, *Halobacterium salinarum*, and *Halobacterium* sp.	Phenanthrene	Soil and liquid medium Aerobic	28, 29, 28, 37, and 22%	Al-Mailem et al., 2017
*Haloarchaea* strains: Ten strains of *Haloferax* sp.	Naphthalene, Anthracene, Phenanthrene, Pyrene, and Benzo(a)anthracene	Hypersaline petroleum produced water (20% NaCl) Aerobic	20–80%	Bonfá et al., 2011
**Ligninolytic fungi**				
*Candida tropicalis* NN4	Indeno[1,2,3-cd] pyrene	Liquid medium Aerobic	91%	Ojha et al., 2019
Fungal mycelia: *Armillaria mellea*, *Pleurotus ostreatus*, *Pleurotus eryngii*, and *Stropharia ferii*	Anthracene and Benzo(a)pyrene	Contaminated soil Aerobic	95 and 50%	Baldantoni et al., 2017
**Non-ligninolytic fungi**				
*Cladosporium* sp. CBMAI 1237	Anthracene, Acenaphthene, Fluorene, Phenanthrene, Fluoranthene, and Pyrene	Liquid medium Aerobic	71, 78, 70, 47, 52, and 62%	Birolli et al., 2018
*Lasiodiplodia theobromae*	Benzo(a)pyrene	Garden soil Aerobic	92%	Wang et al., 2014
**Algae**				
*Selenastrum capricornutum* and *Scenedesmus acutus*	Benzo(a)pyrene	Liquid medium Aerobic	99 and 95%	De Llasera et al., 2016
*Rhodomonas baltica*	Phenanthrene, Fluoranthene, and Pyrene	Liquid medium Aerobic	70%	Arias et al., 2017
**Co-cultures**				
Bacterial–fungal consortium: *Serratia marcescens* L-11, *Streptomyces rochei* PAH-13, and *Phanerochaete chrysosporium* VV-18	Fluorene, Anthracene, Phenanthrene, and Pyrene	100 g soil in pot, Aerobic	98, 66, 90, and 55%	Sharma et al., 2016
Bacterial–algal synergy: *Chlorella* sp. MM3 and *Rhodococcus wratislaviensis* 9	Phenanthrene, Pyrene, and Benzo(a)pyrene	Soil slurry Aerobic	100%	Subashchandrabose et al., 2019

The immobilization of delivering inoculum microbes can serve the solution by increasing shelf life and activities of microbes in the soil system (Mrozik and Piotrowska-Seget, 2010). Huang et al. (2016) reported enhanced pyrene and indeno[1,2,3-cd]pyrene degradation (71 and 81%) in petroleum-contaminated soil using immobilized *Pseudomonas taiwanensis* PYR1 and *Acinetobacter baumannii* INP1 on cinder beads. Immobilization provides biological stability to inoculum microbes, protection from suboptimum substandard environmental conditions, and reduced competition with indigenous microbes (Mrozik and Piotrowska-Seget, 2010). Apart from mesophilic bacteria, bacterial extremophiles such as halophilic, acidophilic, and thermophilic have also been reported for PAH degradation (Table 2). Application of thermotolerant and thermophilic bacteria for PAH degradation is beneficial, as elevated temperature causes increased diffusion of PAHs by decreasing viscosity, ultimately increasing the bioavailability of PAHs (Mehetre et al., 2019). Over the last decade, bacterial community analysis (Muangchinda et al., 2018; Yang et al., 2020), biochemical pathways in bacteria for PAH degradation (Chen et al., 2016; Vaidya et al., 2017), degradation-associated bacterial genes (Peng T. et al., 2018; Sangkharak et al., 2020), enzyme systems (Chen et al., 2016; Wu et al., 2020), and gene regulation of PAH degradation processes (Kan et al., 2020; Wu et al., 2020) have been researched enormously.

#### Archaea

Extreme environmental habitats, particularly saline regions, are most vulnerable to petroleum pollution due to their close connection with oil industries, which mostly release many pollutants, including PAHs, and demand extremophiles rather than conventional microorganisms for bioremediation (Dastgheib et al., 2012). Over the past few years, archaea have drawn the attention of researchers for PAH bioremediation, although few research studies have been reported (Table 2). The degradation pathways and mechanisms behind bioremediation *via* archaea have not been extensively studied like bacteria (Khemili-Talbi et al., 2015). Khemili-Talbi et al. (2015) isolated biosurfactant-producing halophilic arhaeon *Natrialba* sp. C21 from oil-polluted saline water for degradation of phenol, naphthalene, and pyrene at very high salinity conditions (25% NaCl). They also attempted to find the degradation pattern *via* enzyme assays such as catechol 1,2-dioxygenase, catechol 2,3-dioxygenase, protocatechol 3,4-dioxygenase, and protocatechol 4,5-dioxygenase. The maximum activity of catechol 1,2-dioxygenase indicated that degradation occurred *via* the ortho-cleavage pathway.

#### Fungi

Mycoremediation of PAHs has been widely reported in the past several years with numerous fungal species. Unlike bacteria, all fungi do not utilize PAHs as a sole source of carbon; rather, they co-metabolize the PAHs and generate a range of oxidized products including CO_2_. The fungi execute monooxygenase enzyme-mediated PAH degradation (Gupta and Pathak, 2020). Mainly two types, i.e., ligninolytic fungi (white-rot fungi) and non-ligninolytic fungi, have been reported in the literature for LMW and HMW PAH bioremediation (Table 2). Ligninolytic fungi produce enzymes such as lignin peroxidase, manganese peroxidase, and laccases for degradation of lignin present in the wood and simultaneously oxidize the PAHs and convert into diphenol intermediates that eventually oxidize into quinones (Aydin et al., 2017). Ligninolytic enzymes generate water-soluble polar products after catalytic cleavage of aromatic compounds, which are eventually available for fungal metabolism and soil microflora present in the vicinity (Gupta and Pathak, 2020).

On the other hand, non-ligninolytic fungi produce cytochrome P450 monooxygenase-like enzymes, which oxidize the PAHs and lead to form arene oxide and water; further, arene oxides through non-enzymatic rearrangement form phenols, which conjugate with xylose, gluconeric acid, and glucose (Cerniglia and Sutherland, 2010; Ghosal et al., 2016). Some fungal species are also capable of producing biosurfactants in order to overcome the hindrance of less soluble HMW PAHs, which resulted in better degradation (Ojha et al., 2019). Limited studies have been reported on the mechanisms and pathways involved in the breakdown of PAHs through mycoremediation (Aydin et al., 2017; Agrawal et al., 2018). Direct fungi application in the field has many limitations including inadequate biomass growth, huge biomass handling difficulties, lack of application methodologies, and bulk degrading enzyme production, which can be overcome by oxidative fungal enzyme-mediated PAH bioremediation (Harms et al., 2011).

#### Algae

Algae, the primary producers in coastline and estuarine ecosystems, may have a significant role in PAH bioremediation within aquatic ecosystems. Alga-mediated effective PAH removal occurs through cellular biodegradation and/or bioaccumulation (Ke et al., 2010). PAH biodegradation employs both monooxygenase and dioxygenase enzymatic pathways and produces hydroxylated and dihydroxylated intermediates, respectively, depending on the algal type (Chan et al., 2006). Microalgae (Cyanobacteria) are freshwater unicellular green alga, have gained huge attention for its ubiquitous occurrence, easy to propagate, and most prominent efficiency of degrading HMW PAHs (Ke et al., 2010; De Llasera et al., 2016). Few reports on alga-based PAH bioremediation are listed in Table 2. Many alga-based PAH removal studies at the laboratory or microcosm scale have been reported in the literature, although large- or field-scale alga-mediated PAH remediation remains to be uncovered and requires scientific attention to develop successful strategies.

#### Co-cultures

Co-culturing approaches such as bacterial–fungal co-cultures, fungal–algal synergy, and bacterial–algal synergy are proven as the most efficient bioremedial approaches at the laboratory as well as large-scale applications (Sharma et al., 2016; Subashchandrabose et al., 2019; Table 2). During aerobic degradation, algae supply oxygen to enhance degradation. The bacterial–algal synergy is more advantageous over bacterial consortia and bacterial–fungal co-cultures because algae provide various extra polymeric and lightweighted compounds (consist of lipids, proteins, nucleic acids, fermentation products, etc.), which promote bacterial and/or fungal growth and thus enhance PAH degradation (Kuppusamy et al., 2017).

#### Microbial Enzyme-Mediated Bioremediation

Microbial enzyme-mediated bioremediation involves the use of isolated enzymes from bacteria, fungi, and other living organisms for PAH removal. The enzymatic action is extremely efficient and selective due to higher reaction rates and the capability to catalyze reactions at a wide range of temperature and pH. Oxygenase, dehydrogenase, lignin peroxidase, manganese peroxidase, laccases, and phenoloxidases are enzymes responsible for PAH oxidation as mentioned in above subsections (Mohan et al., 2006). The oxidative enzymes from fungi are more efficient because they are less substrate-specific enzymes (Harms et al., 2011; Gupta and Pathak, 2020). Zhang et al. (2020) isolated novel manganese peroxidase gene from *Cerrena unicolor* BBP6 and cloned into *Pichia pastoris*, which had various dye-decolorizing ability along with 80 and 91% of fluorene and phenanthrene degradation activity within 24 h, and the highest recombinant enzyme expression was 154.5 Unit.L^–1^. The only drawback of this method is cost related to production, extraction, and purification of enzymes (Kuppusamy et al., 2017).

### Strategies for Polycyclic Aromatic Hydrocarbon Bioremediation

*Land farming* is a cost-effective and safe treatment for polluted land, in which the native microbiome at a polluted site is stimulated for PAH degradation *via* improving aeration, moisture, and nutrient levels so that the connection of microbes is improved with pollutants and nutrients (Agnello et al., 2016; Kuppusamy et al., 2017). The reduction rate is much higher for LMW PAHs (2–3 rings) than HMW PAHs (4–6 rings), and this method is applied usually for a thin layer of land surface (Das and Das, 2015; Silva-Castro et al., 2015; García-Sánchez et al., 2018). This simple method requires less maintenance, nearly no cleanup obligations, and slight monitoring efforts. Limitations are slow degradation rate after initial rapid degradation rate due to the concentration gradient of pollutants, affected only superficial 10–35-cm accessible soil layer, and largely influenced by surrounding uncontrollable and unintentional conditions like heavy rainfall (Gan et al., 2009).

Natural attenuation method enhances the degradation capacities of innate microbiome by improving aeration, moisture, and nutrient levels. If natural attenuation is performed on polluted land, then it is a good example of land farming method. Chikere et al. (2017) reported 98% total petroleum hydrocarbon and 85% poly aromatic hydrocarbon degradation by enhanced natural attenuation in crude oil-polluted field-scale bioremediation after. The enhanced natural attenuation was processed by nutrient addition (N:P:K ratio 2:1:1), tilling, periodic water irrigation, and intermittent turning of soil to make sure there was uniform aeration (Chikere et al., 2017).

*Biostimulation* is a remediation method in which the activities of indigenous microbes can be encouraged by the addition of nutrients (N,P,S, and K), slow/fast releasing fertilizers, organic wastes, humic acid, and/or terminal electron acceptor. It is basically used to overcome limitations of microbial growth and activities. Different combinations of macro- and micro- nutrients are used to enhance PAH degradation (Das and Das, 2015). Patel et al. (2019) tested NPK fertilizer, urea fertilizer, and AS fertilizer as biostimulating agents to enhance phenanthrene and fluoranthene degradation by mixed bacterial cultures. Biostimulation can also be performed through an adaptation approach in which high pre-exposure of target pollutants are applied for adaptation of selective organisms having the capacity to survive and utilize target pollutants (Mohan et al., 2006).

A new era of nanobiotechnology leads to the development of a more competitive biostimulation approach for rapid PAH remediation based on the use of nanofertilizers and nanominerals, which enable broader distribution of nutrients in deeper soil (Kuppusamy et al., 2017). A time interval study conducted by Bianco et al. (2020) on the effects of anaerobic biostimulation such as digestate, fresh organic fraction of solid municipal waste, and combination of micro-/macronutrients (ratio of soil and biostimulants was 10:1) on the degradation of the four PAH mixture (200 mg.kg^–1^ of anthracene, phenanthrene, pyrene, and fluoranthene) in marine sediment at 37°C and 130 rpm confirmed biostimulation efficiency for PAH degradation (55%) compared to control without any supplementation (12%). The application of digestate and organic waste as biostimulants during degradation signifies the economic perspective as well as encourages the renewable remediation strategy (Bianco et al., 2020). Blood meal is a dark-colored complex non-toxic liquid of animal origin, which acts as a slow releasing fertilizer and is rich with lysine, valine, leucine, tryptophan, and histidine. Recently, biostimulation using blood meal along with weekly soil plowing was studied for *in situ* bioremediation of dichlorodiphenyltrichloroethane (DDT)- and PAH-polluted farmland soil (Wang et al., 2017).

*Composting* is one of the most preferable and cost-effective remediation methods for pollutant degradation in soil, which improves soil organic content and soil fertility, and it is one type of biostimulation in which organic content is added (Chen et al., 2015). Composting remediation is more successful for 3- and 4-ring PAHs than 5- and 6-ring PAHs, as higher ring PAHs may negatively affect the microbial activities of compost and their natural bioavailability was low (Gan et al., 2009; Guo et al., 2020). The compost bulking agents such as spent mushroom, soot waste, agricultural wastes, maple leaves, cow manure, pig manure, activated sludge, etc., can be used in PAH degradation that support the enhancement of microbial population and raise the required temperature for degradation (Mohan et al., 2006; Shah, 2014; Das and Das, 2015). Guo et al. (2020) studied biodegradation of PAHs in polluted sewage sludge by a co-composting method using green forest waste. The experiments were performed with three different rations of sewage sludge and green forest waste. PAH degradation (75.2%) was highest in ratio 3:2, followed by ratio 3:1 (70.7%) and ratio 3:3 (62.4%) after 50 days of composting in compost windrows (1.5 m width × 1.2 m height × 10 m length).

*Bioaugmentation* is the introduction of inoculum of pollutant-degrading single microorganisms or group of microorganisms to achieve optimum degradation and sometimes to improve the catabolic capacities of indigenous microbes (Das and Das, 2015). It is effective, rapid, easily publicly adaptable, easily applicable, and versatile alternative for PAH degradation; nevertheless, unpredictable (Kong et al., 2018). Application of the bioaugmentation strategy for PAH degradation may include bacteria, archaea, fungi, and algae as pure cultures as well as mixed cultures; detailed description is presented in the section *Microbial Remediation of Polycyclic Aromatic Hydrocarbon Pollution* (Ghosal et al., 2016). The degradation of PAHs by microorganisms occurs in the presence of oxygen and in the absence of oxygen, which are called aerobic degradation and anaerobic degradation, respectively. In anaerobic biodegradation, microbes use other substances such as nitrate, sulfate, iron, manganese, and carbon dioxide as electron exchanger during degradation and produce carbon dioxide and methane as the final products. Anaerobic biodegradation is helpful to remediate the deep underground soil where oxygen is absent or very low (Gan et al., 2009).

*Bioreactor* is an *ex situ* controlled system for efficient PAH degradation; addition of non-ionic surfactants, bioaugmentation with useful microbes, and/or biostimulation with additional nutrients enhance PAH bioremediation process in bioreactors (Mohan et al., 2006). Soil column and soil slurry bioreactors degrade effectively the soil-bound contaminants under controlled and optimized conditions. The continuous fed batch reactors (anaerobic-anoxic-aerobic, 5 L each) were proved to be potential for 300 mg.L^–1^ of naphthalene degradation (99%) in influent wastewater from coke oven industry along with sulfate and ammonical nitrogen as biostimulants and cow dung slurry as inoculum by Yadu et al. (2019). Forján et al. (2020) designed a pilot- scale soil slurry bioreactor for PAH-polluted factory soil in which dissolved oxygen (8 mg/L), pH (∼8), and temperature (28°C) probes were constantly controlled. Soil slurry bioreactor was prepared by combined approach of biostimulation (C:N:P ratio of 100:10:1) and bioaugmentation using *Rhodococcus erythropolis*, which reported 89.3, 79.7, 72.0, and 82.1% degradation of 2-ring, 3-ring, 4–6-ring, and total PAHs, respectively, after 15 days of bioreactor process (Forján et al., 2020).

*Phytoremediation* is an *in situ* method in which the plants are used to remove PAHs or to convert them into less harmful components in soil, sediment, surface water, and groundwater (Das and Das, 2015). Plants remediate the organic pollutants by different mechanisms such as phytoextraction (withdrawal of pollutants from soil), phytovolatilization (atmospheric release of volatile pollutants from soil *via* plant organs), and phytodegradation (degradation of pollutants by enzymes released from plant and/or plant-associated microbes) (García-Sánchez et al., 2018). Plants help in soil aeration by increasing permeability and by cracking soil masses, which favor PAH aerobic biodegradation (Gitipour et al., 2018).

During phytoremediation, plants resist easily a range of environmental assaults due to their sessile characteristic. Plants are selected ideally based on their quality to grow at a polluted site and growth time, biomass productivity, ability to support active soil microbial population, capability to degrade pollutants, and capability to adapt to environmental conditions (Cook and Hesterberg, 2013). Economic viewpoint suggests phytoremediation with grass is preferable due to less maintenance, low nutrient requirements, robust growth, tolerance for sought, acidic and, cold conditions, and their very fibrous root system, which may help enhance soil microbial activities (Gan et al., 2009; Cook and Hesterberg, 2013).

He and Chi (2019) investigated phytoremediation capabilities of two submerged aquatic plants, *Vallisneria spiralis* and *Hydrilla verticillata*, in PAH-polluted sediments at pilot scale. The experiment was conducted for 108 days, and results indicated that dissipation of phenanthrene and pyrene was highest in sediment planted with *V. spiralis* (85.9 and 79.1%), followed by sediment planted with *H. verticillata* (76.3 and 64.6%) and unplanted sediment (76.3 and 64.6%). Higher dissipation of phenanthrene and pyrene in planted sediments was due to plant-supported biodegradation and plant uptake (He and Chi, 2019). Phytoremediation of soil polluted by fly ash PAHs using willows of *Salix* × *smithiana* Willd (checked) was used to remove 50.9% PAHs after 3 years of treatment (checked), which was higher as compared to 9.9% ash PAH removal by natural attenuation in soil (Košnáø et al., 2020).

*Rhizoremediation* is one specific subset of phytoremediation, in which plant-associated rhizosphere microorganisms are used for treatment of polluted soils (Das and Das, 2015). Rhizoremediation is more intensive for PAH degradation and a key of successful rhizoremediation is dependent on the appropriate partnership of plant and microbes that have degradation capabilities (Agnello et al., 2016; Eskandary et al., 2017). Plants provide the huge root surface area for microbial growth and remediate pollutants approximately 10–15 m deep in the soil (Bisht et al., 2015). In rhizoremediation, plant roots supply the nutrients for growth and activities of PAH-degrading microbes in the form of carbohydrates, amino acids, flavonoids, and organic acids, whereas microbes compensate by supporting the plants to conquer against stress generated due to pollutants and reduce the phytotoxicity (Bisht et al., 2015; Eskandary et al., 2017). Kong et al. (2018) conducted field-scale PAH degradation study (3 m × 1.2 m, 0.4 m depth, and 5 tons soil) for 175 days in aged polluted soil of 50-year-old coking plants.

Comparison of four different methods indicated that microbe-associated phytoremediation (*Rhodococcus ruber* Em1 associated with *Orychophragmus violaceus*) was superlative among natural attenuation, bioaugmentation (*Rhodococcus ruber* Em1), and phytoremediation (*Orychophragmus violaceus*). *R. ruber* Em1 combined with *O. violaceus* significantly enhanced the removal of 16 PAHs, 54% as compared to 18, 30, and 36% in other methods. The removal of HMW PAHs with 4–6 rings were much greater by microbe-associated phytoremediation methods (55%) as compared to natural attenuation (10%) and phytoremediation (20%) (Kong et al., 2018). García-Sánchez et al. (2018) also compared four different PAH bioremediation approaches *via* pot experiments with 5 kg of aged polluted soil for 180 days. They found a microbe-associated phytoremediation approach using maize plants along with white rod fungi and indigenous microorganisms as the most beneficial for removal of LMW, HMW, and total 16 PAHs as compared to other approaches, i.e., natural attenuation, myco-augmentation using white rod fungi *Crucibulum leave*, and phytoremediation using maize plants (García-Sánchez et al., 2018).

The advantages of phytoremediation and rhizoremediation as compared with other approaches are that they preserve the natural conditions of the soil, energy is derived primarily from sunlight, high level of microbial biomass in the soil can be achieved, and both are cost-effective and environment-friendly methods. The major drawbacks of both methods are the site where plants cannot grow, large land requirement, limited remediation depth, only applicable for low-level polluted site (plant tolerance level), highly dependent on climate and seasonal conditions, disposal of accumulated PAHs from plant parts, unknown effects of biodegradation products, risk for pollutants to enter the food chain, and uncertainty in treatment duration prediction (Gan et al., 2009; Bisht et al., 2015; Gitipour et al., 2018).

*Vermiremediation* is used as individual and combined with microbes or plants for PAH removal from fine soil (pores size < 0.1 μm). PAHs in pores of fine soil are not bioavailable and bioaccessible for degrading bacteria (size 1–10 μm) and plant root hairs (size 15–17 μm). During vermiremediation, burrowing actions of earthworms enlarge the soil pore size; therefore, degrading microbes and plant root can penetrate into the soil, able to grow and finally able to degrade hidden PAHs (Kuppusamy et al., 2017). Earthworms also remove PAHs from the soil by either dermal absorption or intestinal digestion that biotransform or biodegrade into harmless compounds (Sinha et al., 2008). Benefits of vermiremediation include improvement of physical/biological soil quality, excretion of nutritive constituents as vermicasts, and proliferation of beneficial soil microorganisms (Rorat et al., 2017). Earthworms reproduce speedily using less or no energy, which possibly enhance PAH removal in a short time duration, proving vermiremediation to be very cost-effective, eco-friendly, and sustainable (Sinha et al., 2008). The addition of earthworms *Eisenia andrei* in sewage sludge bioreactor after precomposting had led to higher PAH removal (86, 58, and 62% under three different pre-composting processes) after 5 weeks (Rorat et al., 2017). The only drawback of vermiremediation is that it is applicable for low and medium polluted sites, where earthworms are able to survive and grow (Kuppusamy et al., 2017).

### Factors Affecting Polycyclic Aromatic Hydrocarbon Bioremediation

Polycyclic aromatic hydrocarbon bioremediation depends on many factors, including PAH properties, polluted material properties, environmental parameters, and microbial ecology. Abiotic and biotic factors such as temperature, pH, salinity, humidity, nutrient availability, oxygen level, pollutants bioavailability, pollutants concentration, native microflora availability and their degradation capability, microbial substrate specificity, pre exposure of pollutants, production or addition of biosurfactants, and presence of other carbon sources can apparently diverge from site to site that can influence the process of bioremediation (Mohan et al., 2006; Ukiwe et al., 2013; Ghosal et al., 2016). Many factors are interconnected, for example, temperature variation affects PAH solubility and microbial activity (Mehetre et al., 2019). Texture, permeability, moisture content, density, porosity, nutrient quality, pollution aging, organic matter content, microbial density, and microbial diversity of soil/sediment are important factors in the case of soil PAH bioremediation (Chen et al., 2015). The efficiency of microbial remediation is affected by the distribution of pollutants, distribution of microbes, and metabolic capability of microbes (Kong et al., 2018).

The structure of PAHs is one of the deciding factors for selection of degradation methods. Angular arrangement is thermodynamically the most stable configuration of PAHs, but their bay regions are prone to enzymatic degradation; this is the reason for more biodegradation of angular structures than linear and clustered structures. Whereas linear and clustered PAHs are degraded rapidly by photooxidation and chemical oxidation (Abdel-Shafy and Mansour, 2016; Peluffo et al., 2018). As per Clar’s resonance structure rule, aromaticity of PAHs strongly depends on the number of aromatic π-sextets. Aromatic π-sextet rings are more stable, whereas less aromatic rings (rings with minimum number of double bonds) are more reactive for photo-and chemical oxidation. For example, both angular PAH phenanthrene and clustered PAH pyrene have two aromatic π-sextet rings, but phenanthrene has just one and pyrene has two less aromatic rings.

Hence, pyrene degradation is more rapid than phenanthrene by photo/chemical oxidation. This phenomenon is explained by the study of Peluffo et al. (2018), where they reported higher degradation of pyrene (96% of 2,800 mg.kg^–1^) than phenanthrene (91% of 1,200 mg.kg^–1^) in the batch reactor of aged polluted soil at room temperature within 7 days by thermal (65°C for 6 h)-activated persulfate oxidation. Biache et al. (2017) proved that PAH bioremediation was highly dependent on soil type using bioslurry experiments at 28°C in dark and under continuous shaking (120 rpm) with three different types of soil, i.e., former gas plant site soil (76% degradation), former coking plant site soil (34% degradation), and active wood-treating facility site soil (98% degradation).

### Insights From -Omics Approaches Used for Elucidating Polycyclic Aromatic Hydrocarbon Bioremediation

The studies of PAH-degrading microbial community, degrading genes, regulatory genes of degradation, enzyme systems, intermediates and final products of degradation pathways, and the study of indigenous microbial diversity at polluted sites are associated with an actual understanding of PAH bioremediation. Ideal and successful PAH bioremediation design can be strategized based on knowledge of indigenous microbes at polluted sites, their metabolic versatility, and their adaptations toward environmental harsh conditions (Malla et al., 2018). Unfortunately, this particular knowledge is not available easily, which can be gained by the “genomic era,” including culture-independent approaches such as metagenomics (eDNA), metatranscriptomics (eRNA), metaproteomics (eProteins), and metabolomics (eMetabolites) (Desai et al., 2010; El Amrani et al., 2015). The presence of only 0.1–1.0% culturable microbes in the environment is a major drawback of traditional culture-dependent approaches. The remaining 99.0–99.9% are unculturable microbes due to the lack of knowledge of their culturing conditions (Daniel, 2005; Quintero and Zafra, 2016). So, traditional methods limit the complete evaluation of microbial diversity and their functional potential, which can be overcome by metagenomic methods. Advances in next-generation sequencing and associated *in silico* analysis enable addressing metagenomic studies (Quintero and Zafra, 2016).

Metagenomic methods have two distinct approaches: sequence-based and function-based. Sequence-based metagenomic approach is useful to investigate the actual microbial diversity, microbial evolution, microbial interactions, complete degradation pathways, degrading genes, etc. The function-based metagenomic approach is useful for discovering of novel degradative genes (Malla et al., 2018). The functional capabilities of microbial communities can be analyzed at different levels, i.e., metatranscriptomics, metaproteomics, and metabolomics. Metatranscriptomics decipher mRNA profile for up/downregulation of degradative microbes and related genes. Metaproteomics track pollutants induced by proteins by degrading microbes. Metabolomics analyze metabolites generated by degradative microbes to cope up with pollutants (Desai et al., 2010). Metatranscriptomic approaches are not only useful for predicting the functions of abundant members but also are useful for predicting the functions of low-abundance members that comprise as little as 0.05% of the total cell population (Helbling et al., 2012).

Amplicon-based metagenomics by identifying correct microbial taxa as targets help to develop a sustainable frame for precision bioremediation in soil or sediment. Microbial taxa having the pollutant degradation ability and survival capacity at a polluted site can act as biostimulants, whereas microbial taxa having characteristics such as the abundant presence at site, fast growth, easy cultivation, non-pathogenicity, and able to survive and metabolize high concentrations of pollutants can be utilized for bioaugmentation (Redfern et al., 2019). Redfern et al. (2019) identified *Geobacter* spp. as biostimulation targets, whereas *Mycobacterium* spp. and *Sphingomonads* spp. are bioaugmentation targets for creosote-polluted soil using a metagenomic approach along with the Spearman rank correlation statistics (non-parametric test). According to correlation statistics, all positively correlated taxa can be utilized in PAH bioremediation as they are resistant to PAHs and, conversely, negatively correlated taxa are sensitive to PAHs (Redfern et al., 2019). Muangchinda et al. (2018) reported changes in the bacterial communities between original seawater and mixed PAH-degrading consortium SWO (developed from the same sea water) using high-throughput 16S rRNA gene sequencing. They found an increased proportion of Proteobacteria and decreased proportion of Bacteroidetes in consortium SWO along with lower Shannon and Simpson indices. Microbial diversity of the diesel-degrading consortium and key genes for degrading enzymes were studied by 16S rRNA amplicon and shotgun metagenome sequencing, respectively (García-Sánchez et al., 2018).

Zhang et al. (2019) performed interesting research using artificially pyrene-polluted sediment, which covered studies of bacterial synergism, their metabolic profile under pyrene stress, construction of metagenome-assembled genomes (MAGs), and coordination of aromatic hydrocarbon-degrading genes with various metabolic pathways (such as carbohydrate metabolism, ATP synthesis, carbon fixation, nitrogen metabolism, sulfate metabolism, acylglycerol degradation, and Calvin cycle) (Zhang et al., 2019). They found dominance of uncultivable genera ZD0117 and unclassified *Alteromonadaceae* belonging to Gammaproteobacteria after 30 days of pyrene stress, suggesting their important role in degradation. They constructed a total of 56 MAGs belonging to Gamma- and Alphaproteobacteria; many of them were uncultivable and unclassified, among which 20 MAGs showed the presence of PAH degradation genes (Zhang et al., 2019).

Specific PAH-degrading genes, for example, PAH ring hydroxylating dioxygenases (RHDs), can be utilized as biomarkers for characterization of PAH-degrading microbes. Gene-targeted metagenomics approach based on PAH RHDs was proven suitable and promising for PAH-degrading bacterial diversity study in oil-polluted soil and mangrove sediment (Liang et al., 2019). Storey et al. (2018) noticed 10,000-fold higher abundance of PAH RHD genes in phenanthrene-polluted soil as compared to unpolluted soil. In a nutshell, omics techniques are imperative for monitoring the effectiveness and status of bioremediation. Omics techniques answer the important questions related to the fate of augmented and indigenous degrading microorganisms, enumeration of degrading genes, and the role of uncultivable microorganisms, etc. (Quintero and Zafra, 2016). However, omics approaches are still relatively unexplored and practically limited for PAH bioremediation strategies due to hurdles in omics massive data handling and interpretations.

### Considerable Constraints During Polycyclic Aromatic Hydrocarbon Bioremediation

The site assessment and condition optimization for method suitability and satisfactory results are always required for the implementation of bioremediation strategies. Lack of endorsed criteria for evaluating the failure and success of field-scale bioremediation is a matter that requires attention. Operational difficulties arise during implementation of PAH bioremediation strategies from bench to pilot and finally to the field scale, since the lab-scale experiments do not inevitably accurately reflect site-specific applications. Other considerable constraints for successful bioremediation include remediation cost, inappropriate objectives, lack of time management, lack of maintenance and monitoring, etc. Most of the bioremediation approaches used previously have placed more emphasis on removal of parent pollutants rather than measurement of toxicity reduction of the metabolites generated after PAH biodegradation. The R&D funding and commercial investments for bioremediation processes are lagging far behind than other industrial sectors, as PAH bioremediation does not produce high value-added marketable by-products along with degradation. Laws and regulations related to the environment limit the implementation of some bioremediation approaches such as the use of genetically engineered microorganisms.

## New Developments and Emerging Multi-Process Polycyclic Aromatic Hydrocarbon Remediation Approaches

Nanoparticle (NP)-based eco-engineered bioremediation strategy signifies key emerging research fields to deal with pollutants like PAHs from various matrices such as soil, sediment, surface water, and groundwater. The development in NPs demands additional improvement like enhancement of their characteristics through modification in surface properties called “functionalized nanoparticles,” which are able to play multifunctional roles in the bioremediation field (Basak et al., 2020). Bionano-remediation employs a large range of biomolecules as functionalizing agents, mainly enzymes, proteins, DNA, humic acids, and biosurfactants, for remediating many petroleum hydrocarbons including PAHs (Basak et al., 2020). The added advantages of NPs are efficient reactivity and offering higher surface area due to their nano-scale size. The system of (bio)functionalized NPs has nano-adsorptive and catalytic degrading properties in order to remove PAHs from the polluted environment (Basak et al., 2020). Wang et al. (2015) demonstrated benzo(a)pyrene degradation from polluted surface and subsurface soil using silica NPs coated with zwitterionic lipid (1,2-dimyristoylsn-glycero-3-phosphocholine) bilayers.

The lipid-based (bio)functionalized silica NPs provided two methods of remediation: (1) sequestration of benzo(a)pyrene by adsorption and (2) providing colloidal stability necessary for transport to the polluted source (Wang et al., 2015). Shanker et al. (2017) have synthesized iron hexacyanoferrate (FeHCF) NPs (10–60 nm size) of various shapes (hexagonal, rod, and spherical) using plant origin biosurfactant (saponins) extracted from *Sapindus mukorossi* and water as solvent. The properties such as ion exchange, photomagneticity, and electrochromicity encourage the use of FeHCFs as NPs. Under optimized experimental system (pH 7, catalyst 25 mg, PAHs 50 mg/L, and solar radiation), anthracene and phenanthrene degradation was 80–90%, while fluorene, chrysene, and benzo(a)pyrene degradation was 70–80% in water and soil. Moreover, all PAHs were converted into low-molecular weight non-toxic metabolites after treatment. FeHCF NPs acted as good adsorbents and photocatalysts in the study by Shanker et al. (2017).

**FIGURE 2:**
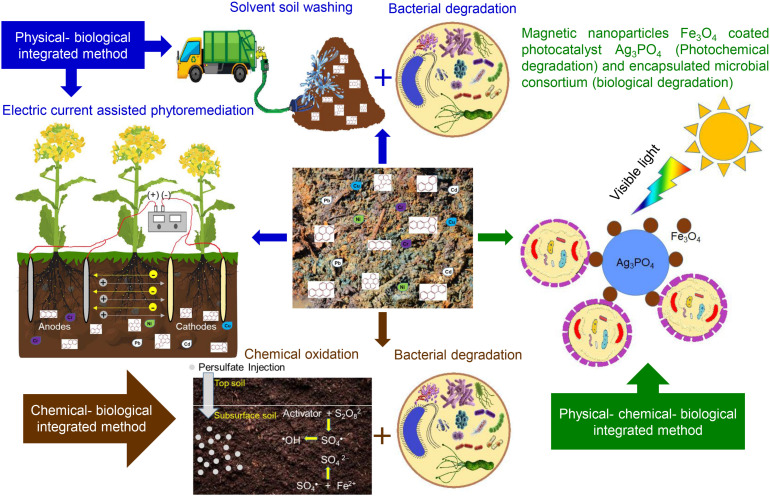
Various integrated approaches used in polycyclic aromatic hydrocarbon (PAH) remediation processes.

Even though the abovementioned physical, chemical, and biological methods are capable of PAH removal, their limitations can be overcome using two or more integrated methods (Kuppusamy et al., 2017). Integrated methods are efficient and advantageous approaches, especially for HMW PAH degradation (Ukiwe et al., 2013; Peng X. et al., 2018). Integrated degradation methods resolve the dead-end product problems and clean the environment completely with less time (Ukiwe et al., 2013). Emerging multi-process/integrated methods for PAH bioremediation include physical–biological coupled remediation (Mao et al., 2017; Cameselle and Gouveia, 2019), chemical–biological coupled remediation (Xu et al., 2019; Gou et al., 2020), multi-biological remediation (Liu et al., 2019; Zeneli et al., 2019), and combination of all three physical–chemical–biological coupled remediation (Wang et al., 2019). Figure 2 summarizes various integrated approaches currently used in PAH remediation.

Mao et al. (2017) have developed *ex situ* surfactant-aided soil washing method using tea saponin (7.5 g/L) and soybean oil (15.0 ml/L) for PAH- and heavy metal-polluted cooking plant soil. Polluted 1 kg soil was washed with 5 L washing solvent in stirring tank at 50°C along with ultra-sonication, and two washing cycles removed 98.2, 96.4, 92.3, 96.3, 94.1, and 89.4% of 3–ring PAHs, 4–ring PAHs, 5–6–ring PAHs, total PAHs, cadmium, and nickel, respectively. They have also reported good efficiency of recovered washing solvent. Further, alkaline solution-based precipitation and inoculation of PAH-degrading *Sphingobium* sp. PHE9 removed 92.6 to 98.4% PAHs from washing solvents of the first and second cycles.

Cameselle and Gouveia (2019) devised an electric current-assisted phytoremediation system for remediation of PAHs and heavy metals from contaminated soil. Fast germinating and growing capabilities of plant species *Brassica rapa* were proven effective among 14 different plant species along with alternating current (AC) from the parallel electrodes. The electric current in the field improved the bioavailability of nutriments and pollutants and favored the growth of plant and rhizosphere bacteria and their remediation ability. The integrated physical and biological methods were proven suitable for the management of mixed polluted soil (Mao et al., 2017; Cameselle and Gouveia, 2019).

In field-scale PAH remediation, chemical oxidation alone may require multiple applications for complete removal of PAHs, which increases the operational cost, and soil quality and soil microorganisms get negatively affected. Similarly, bioremediation alone is not able to degrade HMW PAHs (5–6 rings) from aged and chronically polluted soil due to strong adsorption of HMW PAHs with soil particle. Hence, an integrated chemical and biological method is a cost-competitive and preferable approach, which is able to overcome limitations of either method (Gou et al., 2020). Pre-chemical oxidation reduces soil microbial diversity and richness, so that subsequent PAH biodegradation may be affected, which can be overcome by optimizing the concentration of the chemical oxidant.

Gou et al. (2020) investigated the potential of integrated chemical oxidation using sodium persulfate and anoxic biodegradation in subsurface aged PAH-polluted soil. They monitored the effects of different persulfate dosages (1 and 3% w/w) on abundance and community of soil bacteria and optimized 1% as the suitable dosage. After 180 days of incubation, the integrated approach showed significantly enhanced removal of 3- and 4-ring PAHs from the polluted subsurface soil as compared to chemical oxidation alone and anoxic biodegradation alone. Removal of most of the 5- and 6-ring PAHs occurred by chemical oxidation in the study by Gou et al. (2020). Xu et al. (2019) found that chemical oxidation of benzo(a)pyrene in polluted soil by 20 mmol/L iron-activated sodium persulfate and 10 mmol/L potassium permanganate further enhanced PAH biodegradation up to 98.7 and 84.2% within 60 days.

Liu et al., 2019 have applied two biological approaches together for PAH degradation in aged polluted soil, i.e., bioaugmentation with bacteria *Paracoccus* sp. LXC and biostimulation with humic acid and spent mushroom substrate and have achieved 56.5% PAH degradation. Similarly, multi-process bioaugmentation–biostimulation methods were found to degrade 52 and 87% of total petroleum hydrocarbons and PAHs in refinery solid wastes after 80 days of incubation, which were high as compared to biostimulation alone (47 and 59%) and natural attenuation (37 and 42%) (Zeneli et al., 2019).

Wang et al. (2019) have also developed a novel integrated method in which photochemical degradation and microbial degradation were coupled for multi-PAH remediation in polluted soil. Ag_3_PO_4_ visible light-responsive photocatalyst was attached to Fe_3_O_4_ magnetic NPs and formed active conjugates responsible for HMW PAH degradation in response to photooxidation. Microbial consortium (MC) was immobilized in microcapsules (MI), which was responsible for LMW PAH degradation due to microbial activities. The active photocatalyst conjugate Ag_3_PO_4_@Fe_3_O_4_ was anchored on the membrane of MI embedded with MC, which was called MI-MC-photocatalyst compound system (MCS). MCSs have removed 944.1 mg/kg of PAHs (94.4%) within 30 days under visible light at 25°C along with improvement in soil microbial ecology and decreased soil eco-toxicity, which was confirmed by soil seed germinability (Wang et al., 2019).

## Significance of Polycyclic Aromatic Hydrocarbon Remediation Through Value-Added By-Product Generation

In the current scenario, most of the research studies are drawing attention toward the applicative aspects of by-product generation during PAH biodegradation, e.g., biogas production, bioelectricity generation, biosurfactant production, and extracellular polymeric substance (EPS) production (Jia et al., 2016; Yu et al., 2017; Tripathi et al., 2019; Bianco et al., 2020). Bianco et al. (2020) demonstrated anaerobic degradation of phenanthrene, anthracene, fluoranthene, and pyrene in polluted marine sediments by a biostimulation approach. They supplemented fresh organic residues of municipal solid waste, digestate, and several other nutrients as the biostimulants that are able to degrade a maximum of 55% of total PAHs after 120 days, along with a maximum of 80 ml.gVS^–1^ of bio hydrogen production from 3 to 30 days under acidogenic conditions and 140 ml.gVS^–1^ of bio methane production from 50 to 120 days under methanogenic conditions. Bio methane production showed an increasing trend even after 120 days, suggesting the possibility of higher yield upon prolonged biostimulation (Bianco et al., 2020).

Sayara et al. (2010) developed a composting-mediated PAH degradation approach in contaminated soil under strict anaerobic methanogenic conditions and reported 91% PAH degradation along with 117.9 L of biogas production after 50 days of incubation per kilogram of total solids utilized. Biohydrogen is recognized as a sustainable energy option for fuel cell technology to produce electricity, which minimizes environmental pollution as compared to conventional fuel cell technology. Biohydrogen-based fuel cells can have wide applications like vehicles, home electrical machineries, and other portable batteries (Rahman et al., 2016). Biogas that is generated as a by-product of anaerobic PAH degradation is recognized as a renewable energy source. Biogas in the form of methane is an alternative fuel substitute of fossil fuels and may have applications in energy production due to its capability to generate heat and power (Amin et al., 2011). The applications of biomethane include usage as fuel for stoves/boilers, fuel for engines and gas turbines for producing electricity, fuel for vehicles, and fuel for fuel cells (Sun et al., 2015).

The microbial bioelectrochemical systems contain single or series of chambers in which anode- and cathode-mediated redox reactions are catalyzed particularly by microbes due to activities of electrogenic biocatalysts (Allen and Bennetto, 1993). Electrons generated from the oxidation of organic matter by the microbial action are transferred to the anode *via* conductive material from the cathode, which produce electric current, and electrons at the cathode can be consumed in biotic and abiotic reduction reactions (Logan et al., 2006). In this manner, microorganisms produce bioelectricity during degradation, which can be utilized to fulfill electricity requirements for our resource-intensive society (Kiely et al., 2011).

The biocatalytic reactions improve PAH degradation by providing alternative electron acceptors (Chandrasekhar and Mohan, 2012). Yu et al. (2017) reported degradation of anthracene (54%), phenanthrene (43%), and pyrene (27%) in contaminated soil coupled with 12.1 mWm^–2^ of power generation by associated soil microbial fuel cells (MFCs). Sherafatmand and Ng (2015) developed sediment MFCs, which were able to work in both aerobic and anaerobic PAH degradation conditions. The degradation of naphthalene, acenaphthene, and phenanthrene was 42, 31, and 36% along with 6.0 mWm^–2^ of power generation under aerobic conditions, whereas degradation was 77, 53, and 37% along with 3.6 mWm^–2^ of power generation under anaerobic conditions, respectively (Sherafatmand and Ng, 2015).

Polycyclic aromatic hydrocarbon degradation is also involved in the production of prominently applicable by-products particularly biosurfactants and EPSs. Biosurfactants are amphiphilic biomolecules mainly produced by a wide range of PAH degraders in order to enhance the bioavailability of PAHs and hence accelerate the degradation efficiency (Tripathi et al., 2019). The researchers have reported diverse forms of biosurfactants, mainly rhamnolipids, lipopeptides, glycoproteins, and surfactins from different PAH degraders (Nie et al., 2010; Ibrar and Zhang, 2020; Kotoky and Pandey, 2020; Tao et al., 2020). The chemically diverse biosurfactants are known as greener biomolecules, which have tremendous applicative aspects apart from field-scale bioremediation, including food, agricultural, oil, cosmetic, and pharmaceutical industries (Farias et al., 2019; Jimoh and Lin, 2019). Their multiple worthwhile properties such as higher foaming, low critical micelle concentrations, and high selective estimable surface activity make them excellent dispersing agents, emulsifiers, and promising substitutes of chemically synthesized surfactants (Jimoh and Lin, 2019).

Farias et al. (2019) formulated six mouthwashes using peppermint essential oil, chitosan, and biosurfactants extracted from *Pseudomonas aeruginosa* UCP 0992, *Bacillus cereus* UCP 1615, and *Candida bombicola* URM 3718 for the control of cariogenic oral microorganisms. Validated results revealed that potential application of biosurfactants in mouthwash preparations is safe and a feasible alternative (Farias et al., 2019). Biosurfactants are also applicable for the remediation of heavy metals from contaminated environments apart from hydrocarbon bioremediation (Ravindran et al., 2020). Several researchers demonstrated application of biosurfactants such as rhamnolipid, lipopeptide, and glycoprotein in microbe-enhanced oil recovery (Arora et al., 2019; Purwasena et al., 2019).

Polycyclic aromatic hydrocarbon degraders secrete EPSs for functioning of various vital physiological processes, which include water-retentive protective layer formation around bacterial cell, bacterial adherence to the surfaces, cell aggregation, microbial floc and biofilm matrix establishment, and resistance toward heavy metals (Flemming and Wingender, 2010; Hong et al., 2013). EPSs are produced in the form of exopolysaccharide, alginate, dextran, gellan, pullulan, xanthan gum, cellulose, pectin, starch, mutan, and levan by a diverse range of microorganisms (More et al., 2014). Such structurally and chemically diverse by-products (i.e., EPSs) of PAH degradation have promising applications in multiple areas such as pharmaceutics, healthcare, food, and agriculture due to their gelling, stabilizing, thickening, and emulsifying characteristics (Moscovici, 2015; Wang et al., 2018). Additionally, EPSs are also linked with the remediation of a wide range of pollutants, mainly toxic heavy metals, hydrocarbons, and inorganic pollutants present in wastewater and soil (Shojaipour et al., 2020). The research studies have also demonstrated the role of exopolysaccharides in microbe-enhanced oil recovery (Li et al., 2017).

## Perspective, Future Outlook, and Conclusion

The pollution of bio-toxic PAHs originating from various sources discussed above has severely affected pristine environments and human health. As discussed in this review, considerable progress has been made in developing various technologies and strategies for environmental remediation of PAH pollution. However, many challenges remain unresolved, and site remediation of PAHs is still a formidable task. Laboratory- and pilot/reactor-scale studies have provided ample evidence, detailed and distinct mechanistic insights into LMW and HMW PAH degradation. LMW PAHs were found to be comparatively easy to degrade, while most of the carcinogenic PAHs are HWM PAHs with more compact rings and their degradation is very difficult. Moreover, the accumulated evidence by several years of research has suggested that the replications of lab-scale results into field-scale applications are inadequate. From the scientific literature assessed in this review, it was apparent that the bioremediation approaches provide a plausible solution for PAH remediation. There is a new trend in rejuvenating the conventional treatment systems such as phytoremediation, landfarming, and composting for PAH treatment. The synergetic interactions between microorganisms and plants have been well exploited for *in situ* bioremediation of PAHs. Emerging integrated approaches such as eco-engineered bioremediation of PAH using bionanoparticles, bioaugmentation–biostimulation, photochemical degradation followed by microbial degradation, immobilization of microbial consortium in microcapsules, etc., have greatly enhanced efficiency for remediation of PAH contamination in the environment.

Understanding and recognizing the dynamics of PAH degradation under soil and marine environment are major problems, which need to be addressed in future research. Under the soil ecosystem, the released PAHs often get entrapped in black-clayish-carbon particles and in coal tar, which significantly reduce their bioavailability. Thus, it is one of the major bottlenecks for successful PAH bioremediation. The optimal role of dissolved oxygen concentration, available nutrient, co-contaminants (e.g., heavy metals, phenolics, etc.), and abiotic parameters decides the fate of PAH remediation approaches. Since the natural environment is an interactive and interdependent system for niche and nutrient cycling by microbial communities, an understanding of the synergistic hydrocarbon degradation mechanisms is required for proper implementation and scale-up of *in situ* biostimulation and bioaugmentation approaches. Notably, the newer developments in the generation of value-added by-products (e.g., biofuels, biosurfactants, and bioenergy) during PAH remediation are a pragmatic approach, which need to be explored in future research.

## Author Contributions

DM and KJ conceived the initial theme and concept for the review. AP carried out the literature survey, translated the concept, and wrote the manuscript. KJ and SS contributed in writing parts of the manuscript. CD and DM comprehensively edited, revised, and proofread the manuscript. All authors read and approved the final manuscript.

## Conflict of Interest

The authors declare that this review article compilation was conducted in the absence of any conflict of interest.
